# Not all that glitters is gold: off-target recombination in the somatostatin–IRES-Cre mouse line labels a subset of fast-spiking interneurons

**DOI:** 10.3389/fncir.2013.00195

**Published:** 2013-12-10

**Authors:** Hang Hu, John Z. Cavendish, Ariel Agmon

**Affiliations:** Department of Neurobiology and Anatomy and the Sensory Neuroscience Research Center, West Virginia UniversityMorgantown, WV, USA

**Keywords:** Cre mice, inhibitory interneurons, somatostatin, parvalbumin, fast spiking, Cre reporters

Neurons in the mammalian brain are a highly diverse population with a complex assortment of electrophysiological, morphological and molecular properties, which has hindered efforts to classify them into genetically and functionally meaningful subtypes and to understand their various roles in the normal or pathological brain. Nowhere is this issue more acutely felt than in the study of inhibitory cortical interneurons, whose classification is still a source of contention and confusion (Markram et al., 2004; Petilla Interneuron Nomenclature et al., 2008; Defelipe et al., 2013). A major boon to investigators has been the development of mouse lines in which genetically defined subsets of interneurons express fluorescent proteins, allowing their identification and targeting during electrophysiological recordings or imaging experiments (Oliva et al., 2000; Meyer et al., 2002; Chattopadhyaya et al., 2004; Ma et al., 2006). More recently, investigators funded by the NIH Neuroscience Blueprint project have developed a toolbox of “driver” lines in which the Cre recombinase gene is inserted immediately downstream to genes that are known markers of specific interneuron subsets (Taniguchi et al., 2011). These Cre lines can be bred with mice carrying floxed genes, to generate cell type-specific knockouts of genes of interest. In addition, by breeding these lines with mice from a parallel toolbox of Cre reporter lines in which a gene coding sequence is inserted after a lox-STOP-lox cassette, or by transfecting them with viral vectors carrying similar constructs, investigators can induce cell-type specific expression of any gene of interest, from inert fluorescent proteins to calcium probes and light-activated ion channels or pumps (Madisen et al., 2010, 2012; Zariwala et al., 2012). While these technologies carry great promise and have already enabled some important findings, the rush to use them also carries considerable risk, if the relevant expression patterns are not fully characterized. A case in point is the somatostatin–IRES-Cre (SOM-Cre) mouse line (Taniguchi et al., 2011), in which Cre expression was targeted to cells containing the neuropeptide somatostatin (SOM). In the cerebral cortex, SOM-containing neurons are a well-studied population of dendritic-targeting inhibitory interneurons (Ma et al., 2006; Silberberg and Markram, 2007; Fanselow et al., 2008; Tan et al., 2008; Ma et al., 2010; Fino and Yuste, 2011). The SOM-Cre line has already been used in several high-profile studies, and in most of these the authors tacitly assumed—but did not validate—that Cre-mediated recombination was restricted to SOM interneurons (Adesnik et al., 2012; Gentet et al., 2012; Lee et al., 2012; Wilson et al., 2012; Chiu et al., 2013; Kvitsiani et al., 2013; Xu et al., 2013). We found, however, that 6–10% of neurons expressing a Cre-dependent reporter in any given cortical layer were fast-spiking/ parvalbumin-expressing (FS/PV) interneurons, a subtype quite distinct from SOM interneurons in electrophysiological etc., morphological and molecular properties (Rudy et al., 2011) [Note that there is another SOM-Cre line reported in the literature (Lovett-Barron et al., 2012), which we did not test].

**Figure 1:**
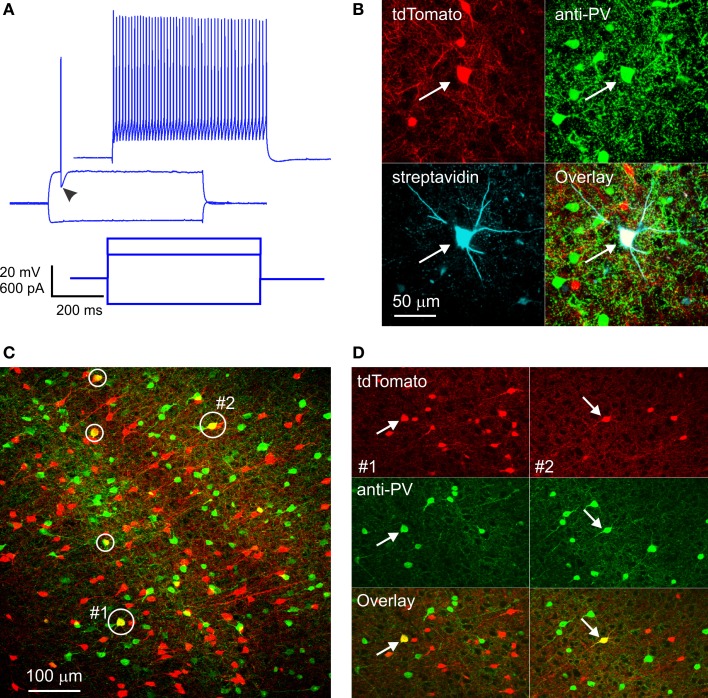
**RFP-expressing fast-spiking interneurons in SOM-RFP mice. (A)** Voltage responses (upper panels) of an RFP-expressing layer 5 barrel cortex neuron to the intracellular current steps shown in the lower panel. Note the pronounced after-hyperpolarization (arrowhead) and the non-adapting spike train, typical of FS interneurons. **(B)** The same neuron was found *post-hoc* to contain biocytin and RFP and to be immunopositive for PV. **(C)** A projection of a confocal stack from cortical area A1, showing five RFP-expressing cells immunopositive for PV (circled). The cells labeled #1 and #2 are shown in **(D)**. **(D)** Single optical sections from subregions of the same field of view as in **(C)**, in the vicinity of cell #1 (left panels, arrows) and cell #2 (right panels, arrows), separated into red and green channels.

Our experiments complied with all relevant institutional and federal animal use guidelines and regulations and were approved by the West Virginia University Institutional Animal Care and Use Committee, and the methods have been previously published (Hu et al., 2011). We crossed SOM-Cre males with females of the Ai14 reporter line (Madisen et al., 2010), to generate double transgenic progeny in which SOM interneurons express td-Tomato, a red fluorescent protein (RFP). We refer to such double transgenics as “SOM-RFP mice.” We conducted whole-cell recordings in brain slices prepared from the somatosensory cortex of SOM-RFP mice, and were surprised to find that 18% (20/112) of RFP-expressing neurons in cortical layers 3 through 5 exhibited an electrophysiological “fingerprint” typical of FS interneurons and clearly distinct from that of SOM interneurons (Beierlein et al., 2003; Hu et al., 2011) (Figure 1A). To verify the subclass identity objectively, we developed a simple non-linear classifier based on three electrophysiological parameters: spike width at half height (SWHH), after-hyperpolarization (AHP) and adaptation ratio (AR), measured as previously described (Ma et al., 2006). Each cell was tested for three conditions: SWHH < 0.26 ms, AHP ≥ 14.5 mV and AR > 0.56, and was classified as FS if at least 2 conditions were true and as SOM otherwise. We first tested this classifier on a “ground truth” dataset of 91 GFP-expressing interneurons from the X94 line (Ma et al., 2006); all but one were classified correctly as SOM interneurons. Since in the X94 line GFP-expressing SOM interneurons have quasi fast-spiking firing properties, separating them correctly from FS interneurons was a stringent test of the classifier. We then tested the classifier on a sample of 15 GFP-expressing interneurons from the G42 line (Chattopadhyaya et al., 2004) and 96 RFP-expressing interneurons from progeny of a PV-Cre line (Hippenmeyer et al., 2005); all 111 interneurons were classified correctly as FS. Finally, we applied the classifier to our sample of SOM-RFP interneurons; 21 interneurons were classified as FS, including the 20 initially identified. Recordings from progeny of SOM-Cre mice bred with a different reporter line [Ai39 (Madisen et al., 2012)] yielded a similar, though smaller percentage of FS interneurons (2 out of 19).

FS interneurons are uniquely characterized by their PV expression (Kawaguchi and Kubota, 1993); we fixed a subset of slices in which we recorded RFP-expressing FS interneurons, stained them with an antibody to PV and with fluorescent streptavidin (to label the biocytin-filled neurons recorded from), and imaged them on a confocal microscope. Out of 7 RFP-expressing FS neurons recovered, five were immunopositive for PV (Figure 1B), substantiating their electrophysiological identification. The remaining two neurons were likely false negatives, due to wash-out of the cytoplasmic PV protein during the whole-cell recording.

Since electrophysiological sampling can be biased (for example, FS interneurons may be more likely to be targeted for recordings because they are typically larger than SOM cells), we sectioned fixed brains from four SOM-RFP mice and dually immunostained them against PV and SOM. Two brains were from third postnatal week pups (the age range used in our recording experiments), and two were from ~1 month old animals. We imaged sections representing five cortical areas (cingulate, M1, S1, A1, and V1) on a confocal microscope and counted RFP+, PV+, and RFP+/PV+ double-labeled cells in confocal stacks, verifying that double labeled cells were indeed so in single optical sections (Figures 1C,D). In total, about 18,000 RFP-expressing neurons were examined. Of these, on average 6% were immunopositive for PV. This number is a lower estimate, because weakly double-labeled cells (cells with fluorescence intensity in either channel weaker than the average intensity of single-labeled cells in the same optical section) were not counted as double-labeled. When averaged by layer, cortical area and age group, percentage of RFP+/PV+ double-labeled cells was highest in layer 4 of S1, reaching 14% in one animal and averaging 11.5 and 10% in the younger and older animals, respectively. Percentage of double-labeled cells was somewhat lower in other cortical areas and layers, with the lowest (2%) found in layers 2/3 of V1 of the younger animals, but in the older animals we observed 6–10% double labeled cells in all areas and layers except layers 2/3 of V1 (3%). We carefully examined all RFP+/PV+ double-labeled neurons for potential SOM expression, but only 0.4% of them appeared to be immunopositive for SOM. Finally, in the two brains with the best SOM immunostaining (one from each age range) we also examined RFP+/PV− cells for SOM immunolabeling, and found that 8% of RFP-expressing neurons, on average, were immunonegative for both SOM and PV. To what neuronal subtype these immunonegative neurons belonged remains to be determined; however, some of them could have been false-negative for PV, suggesting that the fraction of FS/PV interneurons in these animals could have been higher than our estimate, closer to the percentage observed in our electrophysiological recordings.

Our observation of RFP expression in FS interneurons cannot be explained by leaky expression of the RFP gene (i.e., expression in cells that did not undergo recombination) or by non-specific expression of the Cre gene (i.e., expression not under the control of the endogenous SOM promoter), because we would then expect to see widespread RFP expression in excitatory neurons, which are the majority cortical cell type. Thus, RFP expression must be under the same genetic control as the endogenous SOM gene; however, we found no SOM expression in the subset of double-labeled RFP+/PV+ neurons, consistent with previous studies which observed no overlap between SOM and PV protein expression in mouse and rat cortical interneurons (Gonchar and Burkhalter, 1997; Xu et al., 2010). This apparent paradox can be explained in two different ways. First, both SOM and FS/PV interneurons are born from embryonic progenitors in the medial ganglionic eminence (Batista-Brito and Fishell, 2009), and it is possible that a subset of progenitors (or of post-mitotic neuroblasts) destined to become FS/PV interneurons transiently express SOM. In the SOM-Cre mice these cells will also transiently express Cre recombinase, undergo Cre-mediated recombination and then express RFP for life, even after losing their SOM expression and attaining their mature FS phenotype. Alternatively, it has been reported that a subset of adult mouse cortical interneurons co-express PV and SOM mRNA (Lee et al., 2010). In the SOM-Cre line these neurons will co-express PV mRNA and the bicistronic SOM-IRES-Cre transcript. It is possible that the Cre transcript is translated into protein even though the SOM transcript is not, or that both transcripts are translated but at very low levels, sufficient for Cre-mediated recombination but not for detection of SOM protein.

The two alternative mechanisms above imply slightly different risks to investigators: the first implies that recombination in FS interneurons is expected when breeding SOM-Cre mice with Cre reporter lines, but not necessarily when transfecting adult neurons with viral vectors, while the second mechanism would result in off-target recombination regardless of the mode of delivery of the reporter construct. Either way, our findings underscore an important caveat for researchers using “subtype specific” mouse driver lines, including those in which the Cre coding sequence is presumed to be under the control of the endogenous gene promoter—these lines should be used with caution and with proper validation. Most studies using the SOM-Cre line appear to use it without validation; an exception is a recent study that tested recombination specificity and found that 5% of recombined neurons in the visual cortex were immunopositive for PV (Pfeffer et al., 2013). We found, by a lower estimate, 6–10% off-target recombination in FS interneurons in most cortical areas and layers. When using the SOM-Cre line to express optogenetic constructs for activation or silencing of SOM interneurons, this degree of contamination by FS interneurons can potentially affect the results and lead to erroneous conclusions. For example, a recent study Kvitsiani et al. (2013) observed that about one third of SOM-Cre interneurons tagged by Cre-dependent channelrhodopsin (ChR2) exhibited fast spike waveforms and high firing rates reminiscent of FS interneurons; but the possibility that some of these were actually ChR2-expressing FS interneurons was not considered. We submit that investigators ignoring the potential for off-target recombination when using the SOM-Cre line, or indeed any other Cre driver line that has not been fully characterized, are doing so at their own risk.
